# A systematic scoping review of the methodological approaches and effects of pesticide exposure on solitary bees

**DOI:** 10.1371/journal.pone.0251197

**Published:** 2021-05-14

**Authors:** David M. Lehmann, Allison A. Camp

**Affiliations:** 1 Center for Public Health and Environmental Assessment (CPHEA), Health and Environmental Effects Assessment Division, Integrated Health Assessment Branch, US - Environmental Protection Agency, Research Triangle Park, Durham, North Carolina, United States of America; 2 ORISE Researcher, Oak Ridge Associated Universities, Research Triangle Park, Oak Ridge, North Carolina, United States of America; University of Alberta, CANADA

## Abstract

**Background:**

Pollination services provided by solitary bees, the largest group of bees worldwide, are critical to the vitality of ecosystems and agricultural systems alike. Disconcertingly, bee populations are in decline, and while no single causative factor has been identified, pesticides are believed to play a role in downward population trends. The effects of pesticides on solitary bee species have not been previously systematically cataloged and reviewed.

**Objectives:**

This systematic scoping review examines available evidence for effects of pesticide exposure on solitary bees to identify data gaps and priority research needs.

**Methods:**

A systematic literature search strategy was developed to identify and document reports on solitary bee pesticide exposure-effects investigations. Literature was subsequently screened for relevance using a Population, Exposures, Comparators, and Outcomes (PECO) statement and organized into a systematic evidence map. Investigations were organized by effect category (lethal effects on immatures, lethal effects on adults, sublethal effects on immatures, and sublethal effects on adults), species, pesticide class, and publication year.

**Results:**

A comprehensive literature search of Web of Science and ProQuest Agricultural & Environmental Science supplemented by targeted internet searching and reference mining yielded 176 reports and publications for title and abstract screening and 65 that met PECO criteria (22 included lethal and 43 included sublethal effects endpoints). Relevant design details (pesticide, test compound configuration, study type, species, sex, exposure duration) were extracted into literature inventory tables to reveal the extent endpoints have been investigated and areas in need of additional research.

**Conclusions:**

Evidence mapping revealed diversity in the pesticides and endpoints studied across the database. However, dilution across bee species, lack of complementary laboratory work and paucity of replicated investigations complicate efforts to interpret and apply available data to support pesticide risk assessment.

## Introduction

**Fig 1:**
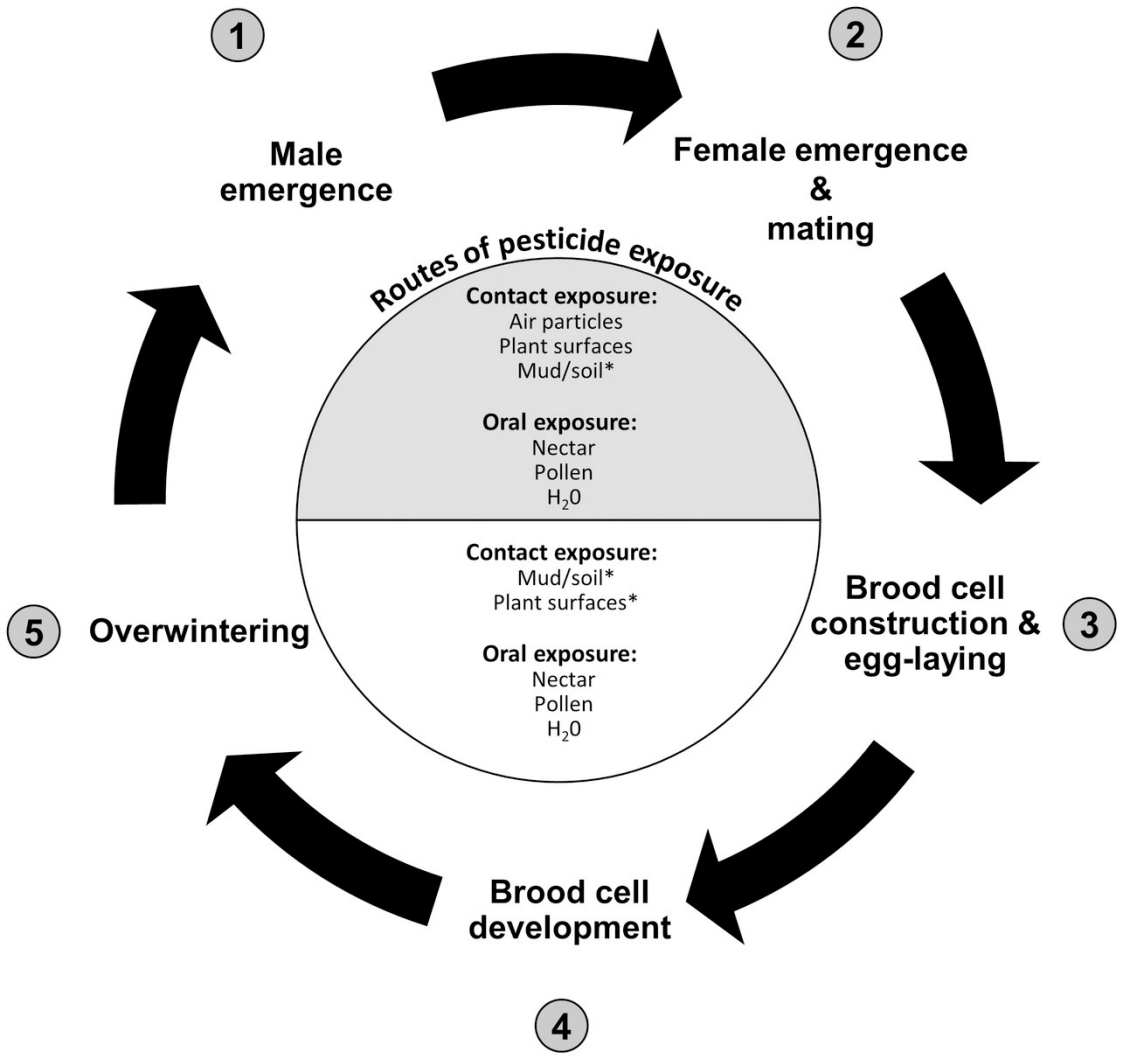
Diagram of the solitary bee lifecycle and potential for pesticide exposure. (1) The solitary bee lifecycle begins when adult male bees emerge from diapause in spring/summer. (2) Females emerge second, mate, and, without help from their conspecifics, (3) construct their nests either underground or in cavities using mud, soil and leaves. Nests are composed of multiple brood cell chambers that are each provisioned with a ball of pollen mixed with nectar upon which the female lays an egg before sealing the brood cell. (4) After hatching, the larvae feed on their mass provisions and continue to develop. (5) Depending on the species, solitary bees overwinter as either prepupae (i.e., non-feeding larva in the 5^th^ instar inside a cocoon) or pre-emergent adults. The cycle repeats when adults eclose from their cells ready to mate the following spring or summer in synchrony with their host plant(s). However, some species (e.g., *Nomia*. *melanderi*, *Megachile*. *rotundata*) may produce a first generation of summer-emerging bees and additional full and partial generations during the same growing season. Depicted in the central circle are potential routes of pesticide exposure for adults (gray section) and egg/larvae (open section). *Relevance of route of exposure dependent on life history of individual solitary bee species.

About 70% of the more than 20,000 bee species worldwide are solitary bees [1]. In contrast to social bees, solitary bee females emerge as adults after winter diapause to build, provision, and lay eggs in nests over several weeks without adult cooperation (although some cooperation is observed in facultatively social species). (Fig 1). Solitary bees provide critical pollination services in both natural habitats and agricultural systems. The life cycles of many species of solitary bees are synchronized to coincide with the flowering patterns of their host plants. The importance of plant-pollinator interaction networks cannot be understated; they form the foundation of entire ecosystems providing feed and habitat for countless other species [2–4]. In addition to being keystone members of natural ecosystems, solitary bees provide valuable pollination services in agricultural systems. Owing to their morphology and proportionally greater direct interactions with flower stigmas, solitary bees are often more efficient pollinators than honey bees and bumble bees [5–9]. Pollination services provided by solitary bees have been shown to significantly drive yield in sunflower [10] and apple [11] crops. For example, on a per bee basis, the Japanese hornfaced bee (*Osmia cornifrons*) can pollinate up to 80% more apple flowers than worker honey bees [12].

Alterations in the abundance and diversity of pollinators is of great concern globally. While most research has been directed at honey bees and, to a lesser extent, bumble bees, populations of some solitary bees are also in decline. A recent report by the Center for Biological Diversity concluded that 1) more than half of the 1,437 native bee species in North America with sufficient data to assess are declining, 2) nearly 25% of native bee species are imperiled and at risk of extinction, and 3) it is likely that species with insufficient population data are also in decline [13]. To quantify changes in network structure and local bee diversity, Burkle and colleagues [3] reviewed insect–plant visitation records collected by Charles Robertson in Illinois, USA in the 1800s and compared them to surveys of the same area collected in 2009 and 2010. They reported dramatic alterations in interaction network structure and function and loss of 50% of bee species. These findings are not limited to the USA. The European Red List of Bees reported that 9.2% of European bee species are threatened with extinction and 37% are in decline [14]. Disconcertingly, the number of bee species found each year in the Global Biodiversity Information Facility has declined since the 1990s, and approximately 25% fewer species were found between 2006 and 2015 than before 1990 [15]. Furthermore, data from the UK indicate that 33% of bee and hoverfly species have declined in their range since 1980 [16]. Solitary bees face many of the same challenges as honey bees and bumble bees including habitat loss, poor nutrition, pests, pathogens and pesticide use [17–19].

Pesticides are widely used in agricultural settings to control an array of arthropod pests, pathogens and weeds. The risk that pesticides pose to bees is assessed using honey bees as a model organism [20]. However, for a multitude of reasons, reliance on the honey bee as a surrogate for risk assessment is being questioned [21–23]. Franklin and Raine [23] recently reported that many studies used to support neonicotinoid registrations do not have large enough sample sizes to confidently report an absence of effect. Furthermore, due to differences in phenology, life history and pesticide sensitivity between solitary bees and honey bees [21, 22], the honey bee may not accurately predict the effects of pesticide exposure on solitary bees [1]. Like honey bees, solitary bees can be exposed to pesticides when foraging during product application (spray and dust) and through ingestion of contaminated pollen and nectar [24]. Notably, honey bee worker brood consume beebread (aged pollen and nectar) through development and for the first two weeks of adulthood and subsequently primarily consume honey (enzymatically processed nectar) [25, 26]. Storing and processing of pollen and nectar has the potential to reduce the pesticide content of provisions [27, 28]. Solitary bees, by contrast, consume fresh, unprocessed pollen and nectar for the entirety of their growth and development [29], and, therefore, may be exposed to greater quantities of pesticides throughout their lifespan.

Due to their life histories, solitary bees can also be exposed to pesticides through other unique routes of exposure (Fig 1; reviewed in [1]). In contrast to honey bees, ground nesting species like *Nomia melanderi* and *Eucera pruinosoa* can be exposed to soil-bound pesticide residues when excavating their nests [30, 31]. However, exposure through contaminated soil is not limited to ground nesting species. Cavity nesting species (e.g., *Osmia* spp.) use soil to build partitions between brood cells [32]. Many species of *Megachile*, *Osmia* and some other solitary bee species incorporate plant leaves into their nests. Consequently, these species may be exposed when processing and transporting pesticide-contaminated leaves [30]. Developing offspring may also come into direct contact with pesticide residues that leach out of contaminated soil surrounding the brood cell and from nesting materials [1, 33].

The effects of pesticide exposure on solitary bee health has not been systematically reviewed previously. Focusing on species commercially used for crop pollination that could be used as surrogates for risk assessment (i.e., *M*. *rotundata* [(Fabricius, 1793), Megachilidae], *N*. *melanderi* [Cockerell, 1906, Halictidae], *O*. *bicornis/rufa* [(Linnaeus, 1758), Megachilidae], *O*. *cornifrons* [(Radoszkowski, 1887), Megachilidae], *O*. *cornuta* [(Latreille, 1805), Megachilidae], *O*. *lignaria* [Say, 1837, Megachilidae] [1], and E. *pruinosa* [(Say, 1837), Apidae], we systematically map and discuss the scientific literature investigating the effects of pesticide exposure on solitary bees. Evidence mapping is an emerging tool to find and categorize existing literature to create a searchable evidence map to identify knowledge gaps and high-priority research needs [34]. Specifically, we catalogue the lethal effects of pesticides on immature and adult bees and the sublethal effects on foraging and nesting activity, larval development and emergence from overwintering, and immunocompetence. We also catalogue pesticide effects on wild bee populations. Knowledge gaps not addressed in the current literature and suggestions for future research are discussed. Overall, our results emphasize the need for future studies examining links between pesticide exposure and its subsequent lethal and sublethal impacts on these important animal pollinators.

## Materials and methods

### Literature search

Our methodology adheres to the guidelines for accurate and transparent health estimates reporting, such as the Preferred Reporting Items for Systematic reviews and Meta-Analyses guidelines (PRISMA) [35]. We searched Web of Science and ProQuest Agricultural and Environmental Science Database to find reports and publications from January 1, 1970 through May 31, 2020. The search strings (“solitary bee” or “solitary bees” or “osmia lignaria” or “orchard mason bee” or “blue orchard bee” or “Osmia cornifrons” or “horned-face bee” or “Japanese orchard bee” or “Osmia bicornis” or “Osmia rufa” or “red mason bee” or “Osmia cornuta” or “European orchard bee” or “Megachile rotundata” or “alfalfa leafcutting bee” or “Nomia melanderi” or “alkali bee”) and (pesticide or pesticides or insecticide or insecticides or fungicide or fungicides or herbicide or herbicides or toxicology) and (exposure or “pesticide exposure” or “acute exposure” or “chronic exposure” “sub-lethal exposure” or “sublethal exposure” or sublethal or “laboratory study” or “semi-field study” or “field study”) were used.

To expand and update our database we completed a second literature search to find reports and publications from January 1, 1970 through the present (search completed on February 11, 2021) using the search terms (Eucera or "Eucera pruinosa" or “Eucera Peponapis pruinosa” or "hoary squash bee") or (("leaf cutter bee" or leafcut* or "leaf-cut*" or "leaf cut*") NEAR/10 bee)) and ((pesticide or pesticides or insecticide or insecticides or fungicide or fungicides or herbicide or herbicides or toxicology)) and ((exposure or "pesticide exposure" or "acute exposure" or "chronic exposure" or "sub-lethal exposure" or "sublethal exposure" or sublethal or "laboratory study" or "semi-field study" or "field study")). The leaf cutter search terms were specifically searched to return results with “bee” within ten words to eliminate results about other leaf cutting insects like attine ants.

Targeted internet searches were performed during March to June 2020 and during February 1 and February 15, 2021 using combinations of various key words including solitary bees, wild bees, native bees, orchard bees, leaf cutter bees, leafcutter, leaf-cutter, leafcutting, *Osmia*, *Megachile*, *N*. *melanderi*, *E*. *pruinosa*, pesticides, insecticides, fungicides, herbicides, toxicology, acute toxicity, chronic toxicity, sublethal toxicity. Additional publications were identified by mining the references from previously identified literature.

### Literature screening

Literature was screened in a two-step process. In step 1, a manual title and abstract review was performed by two independent reviewers to identify records that appeared to meet the PECO criteria [36] (Table 1). Each report and publication was assigned to one or more of the following bins: Relevant to Pesticide Effects on Solitary Bees, Relevant to Pesticide Effects on Wild Bee Populations, or Not Relevant. Supporting supplemental information was also tracked including reports not subjected to peer-review (e.g., theses, conference abstracts, posters, and symposium summaries), conference proceedings, literature reviews, agency reports, risk assessments, and non-English language reports and publications. Information in this category was used to support concepts and discussion of research gaps.

**Table 1:** Populations, Exposures, Comparators, Outcomes (PECO) criteria.

PECO element	Evidence
Populations	Solitary bee species (*M*. *rotundata*, *N*. *melanderi*, *O*. *bicornis*, *O*. *cornifrons*, *O*. *cornuta*, *O*. *lignaria*, *E*. *pruinosa*) of any life stage (including egg, larvae, pupae, and adult stages).
Exposures	One or more oral, inhalation (aerosol, vapor, or particle), or contact treatment(s) with any pesticide (insecticide, fungicide, or herbicide) formulation or technical grade active ingredient administered alone or in combination with one or more other pesticide(s) or pesticide adjuvant/surfactant to a solitary bee species defined above (*in vivo*).
Comparators	When conducted under laboratory or semi-field conditions, a concurrent control group exposed to vehicle-only treatment or untreated control or comparisons across an exposure gradient or between time points following initiation of exposure. For field studies, a concurrent reference plot in a similar landscape with bees placed/monitored a comparable distance from the plot that was not treated with the test pesticide during the period of assessment.
Outcomes	Any examination of primary data on survival, development, eclosion/overwintering, longevity, foraging, food consumption, nesting, learning/memory, physiology, sensory response, immunocompetence, flight/navigation, and/or wild bee abundance.

In step 2, records that were not excluded based on the title and abstract advanced to full text review to confirm eligibility according to the PECO criteria. In addition to confirming PECO-relevance, the effects investigated in each report/publication were identified and organized into the following bins: Lethal Effects on Immatures, Lethal Effects on Adults, Sublethal Effects on Immatures, Sublethal Effects on Adults, and Wild Bee Population Effects. These effect categories were chosen because of their potential to inform risk assessment and would be of interest to regulators, scientists, and stakeholders alike. Reflecting the objective of this systematic review (i.e., to scope the literature and to identify data gaps) and eliminate bias, all studies that satisfied the PECO were included in the synthesis.

To complete the literature screening process, inventories were created for each effect category to develop sortable lists of relevant literature. To maximize the utility of the inventory, study design information (e.g., species, sex, life stage, study design, endpoints evaluated) was extracted (confirmed by second reviewer) for each report/publication in Microsoft Excel.

## Results

### Literature search results

**Fig 2:**
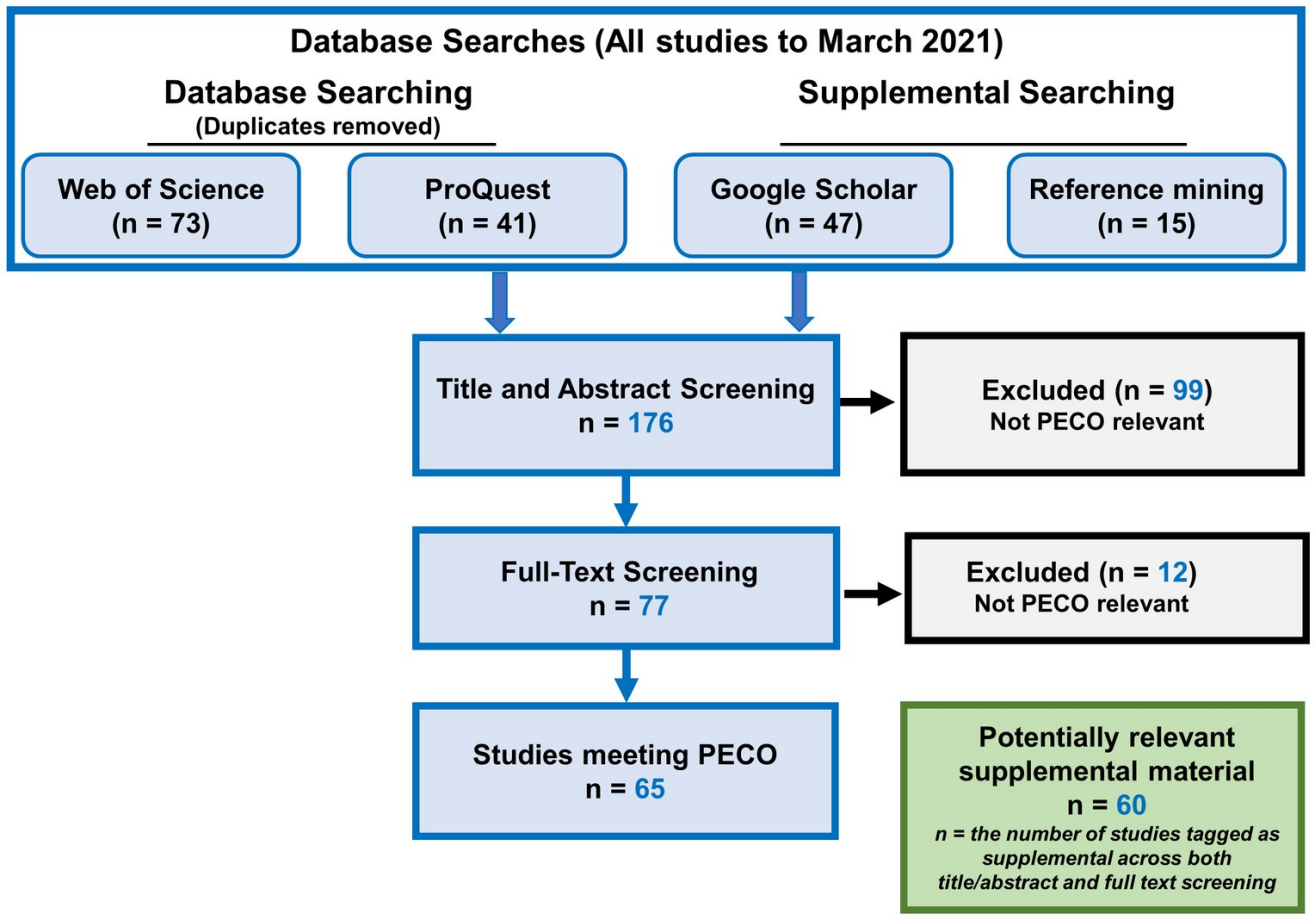
Literature search and selection flow. The study flow diagram describes the number of publications processed at each step of the evaluation. Literature searches for Web of Science and ProQuest were conducted from January 1, 1970 to February 2021. Targeted internet searches were performed using Google Scholar during March and May 31, 2020 and February 1 and February 15, 2021. In total, 65 publications containing solitary bee pesticide exposure-effects investigations were identified. Since the objective of this systematic review was to scope the literature and identify data gaps, all PECO-relevant studies were included in the sections that follow.

**Fig 3:**
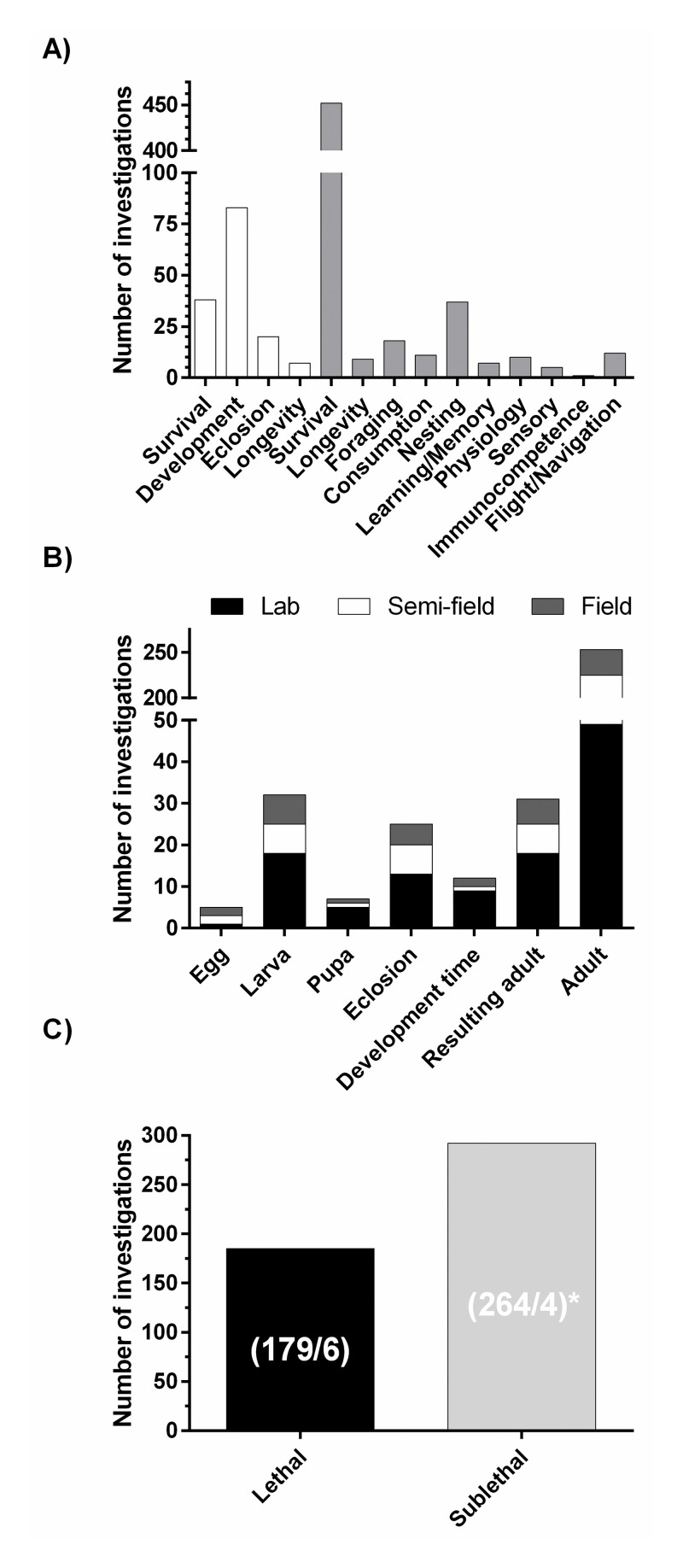
The number of insecticide, fungicide, and herbicide investigations identified for this review. (A) The number of investigations identified for various effect types. Open bars signify investigations performed in immature bees or resulting adults. Filled bars signify investigations performed with adult bees. (B) The number of investigations identified for solitary bee life stages. The number of investigations for each life stage conducted under lab, semi-field and field conditions are stacked to produce the total number of investigations conducted. (C) The number of investigations identified for lethal and sublethal endpoints. The number of lethal investigations performed in adults and immatures and the number of sublethal investigations evaluating field realistic and non-field-realistic concentrations are shown in parenthesis, respectively. *Twenty-four investigations did not report whether or not they used field-realistic concentrations or non-field-realistic concentrations.

**Fig 4:**
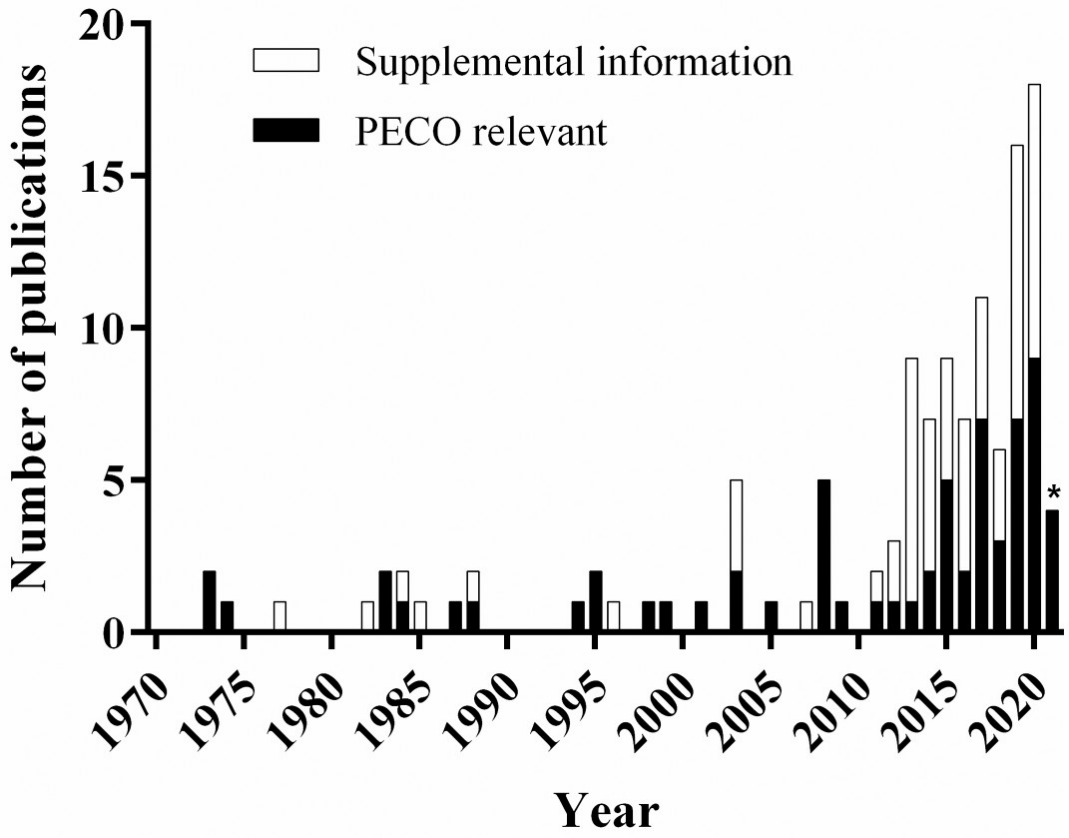
Number of solitary bee publications by year. Web of Science, ProQuest Agricultural and Environmental Science Database, and Google Scholar were searched to February 11, 2021. All results were compiled and organized by year of publication. The number of PECO relevant publications are shown in the black portion of the bar. Publications containing supporting supplemental information are shown in the open portion of the bar. *Since our search was completed prior to the release of all 2021 publications, not all publications for the year 2021 were available for this systematic evidence map.

The PRISMA report describing the minimum set of items required to be reported for a systematic review is provided in (S1 Table in S1 File). The results of the literature search and screen are summarized in Fig 2. A total of 176 reports and publications were subjected to manual title and abstract review for relevance (S2 Table in S1 File). Of these, 77 were identified as potentially relevant and were subjected to full-text review and categorization by effect categories. Finally, 65 PECO relevant publications were included in literature inventories organized into 5 major bins (i.e., Lethal Effects on Immatures, Lethal Effects on Adults, Sublethal Effects on Immatures, Sublethal Effects on Adults, and Wild Bee Population) that encompassed 14 effect categories with survival and development being the most frequently studied outcome of pesticide exposure in adults and immature bees, respectively (Fig 3A). PECO relevant publications were published in 36 different journals. The earliest were conducted in the year 1973 (Fig 4). Adults are the most frequently studied life stage (Fig 3B). Except for studies concerning the adult life stage, laboratory investigations are most common (Fig 3B).

### Distribution of investigations across species

**Fig 5:**
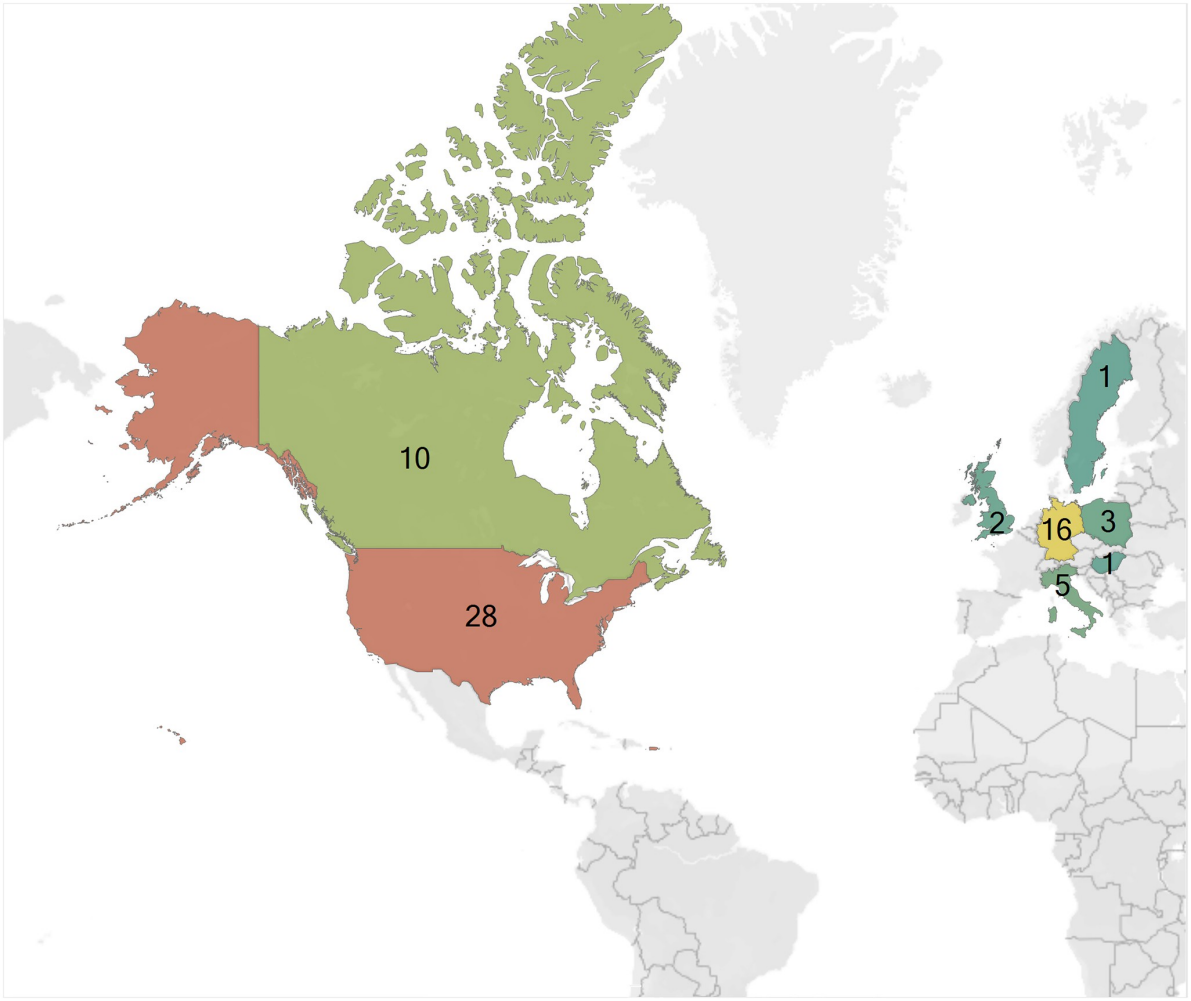
Worldwide distribution of solitary bee studies. The number of publications completed in each country is shown numerically and increases along a color gradient from green to red. This figure was constructed using software developed by Tableau Incorporated (www.tableau.com). The world map was used under a CC BY-SA copyright from OpenStreetMap contributors (www.openstreetmap.org/copyright).

The number of publications investigating the effects of pesticides on solitary bees has been increasing over the last decade (Fig 4). Among these publications, 37 investigated *Osmia* spp., 28 investigated *Megachile* spp., 9 investigated *N*. *melanderi* and only 1 investigated *E*. *pruinosa* (Table 2). Solitary bees used to support these investigations were sourced from the U.S.A. (43.8%) and Europe (40.6%). Investigations were also performed using bees sourced from Canada (14.1%), and one investigation (1.6%) did not specify the source of the bees (Fig 5, S2 Table in S1 File).

**Table 2:** The number of publications investigating the effects of pesticides on commercially available solitary bee species.

Bee species	Number of publications*
*M*. *rotundata*	28
*O*. bicornis**	22
*N*. *melanderi*	9
*O*. *lignaria*	8
*O*. *cornuta*	5
*O*. *cornifrons*	2
*E*. *pruinosa*	1

*Individual publications may have investigated more than one species of solitary bee.

**Includes one published report involving *O*. *rufa* (renamed *O*. *bicornis*).

### Pesticides studied in solitary bees

Most reports investigated the effects of insecticides (52 publications), fungicides (13 publications) and herbicides (2 publications) (Tables 3–5, S3 Table in S1 File). Even though combinations of pesticides are commonly applied in practice, most available reports studied compounds individually. Only 20 of the 65 exposure-effects publications identified in this review investigated the effects of mixtures of two or more pesticides (Table 6; S3 Table in S1 File).

**Table 3:** Insecticides studied in solitary bees. The number of investigations that used a formulation or technical grade active ingredient alone are shown in parenthesis.

Insecticide class	Bee species	Number of publications*	Number of investigations (in formulation, technical grade)
Anthranilic diamide	*M*. *rotundata*, *O*. *bicornis*, *E*. *pruinosa*	3	4 (3, 1)
Benzoylurea	*M*. *rotundata*, *N*. *melanderi*, *O*. *bicornis*	4	9 (7, 0)**
Biopesticide	*O*. *bicornis*, *O*. *cornuta*	3	13 (1, 8)**
Butanolide	*M*. *rotundata*, *O*. *bicornis*	2	3 (3, 0)
Carbamate	*M*. *rotundata*, *N*. *melanderi*, *O*. *bicornis*	7	81 (46, 30)**
Chlorinated hydrocarbon	*M*. *rotundata*, *N*. *melanderi*	1	2 (0, 0)**
Formamidine	*M*. *rotundata*, *N*. *melanderi*	1	2 (0, 0)**
Macrocyclic lactone	*O*. *lignaria*	1	4 (0, 0)*
Neonicotinoid	*E*. *pruinosa*, *M*. *rotundata*, *N*. *melanderi*, *O*. *bicornis*, *O*. *cornifrons*, *O*. *cornuta*, *O*. *lignaria*	27	36 (8, 22)**
Organochloride	*M*. *rotundata*, *N*. *melanderi*	3	6 (0, 2)**
Organochlorine	*M*. *rotundata*, *N*. *melanderi*	2	8 (0, 2)**
Organophosphate	*M*. *rotundata*, *N*. *melanderi*, *O*. *bicornis*, *O*. *cornifrons*, *O*. *lignaria*	17	155 (29, 37)**
Organosulfur	*M*. *rotundata*, *N*. *melanderi*	1	5 (0, 0)**
Oxadiazine	*O*. *bicornis*	1	1 (0, 1)
Phenylpyrazole	*M*. *rotundata*, *N*. *melanderi*	1	2 (0, 2)
Pyrethroid	*M*. *rotundata*, *N*. *melanderi*, *O*. *bicornis*, *O*. *cornifrons*, *O*. *lignaria*	13	38 (11, 4)**
Spinosyn	*M*. *rotundata*, *N*. *melanderi*, *O*. *bicornis*, *O*. *cornifrons*, *O*. *lignaria*	4	8 (3, 5)
Tetramic acid	*O*. *cornuta*	1	1 (0, 1)

For the purposes of this review, an investigation is defined as one or more PECO-relevant outcomes resulting from pesticide exposure (i.e., an investigation reporting multiple outcomes equates to one investigation).

*Individual publications may have investigated more than one species of solitary bee and more than one class of insecticide.

**Count excludes investigations that did not clearly specify if the test compound was technical grade or a commercial formulation.

**Table 4:** Fungicides studied in solitary bees. The number of investigations that used a formulation or technical grade active ingredient alone are shown in parenthesis.

Fungicide class	Bee species	Number of publications*	Number of investigations (in formulation, technical grade)
Benzimidazole	*M*. *rotundata*, *O*. *lignara*	3	3 (3, 0)
Carboxylic ester	*O*. *cornuta*	1	1 (1, 0)
Dicarboximide	*M*. *rotundata*, *O*. *lignaria*	4	4 (4, 0)
Metal	*O*. *bicornis*, *O*. *cornuta*	3	7 (1, 6)
Metalloid	*O*. *bicornis*	2	5 (0, 5)
Phthalimide	*M*. *rotundata*, *O*. *lignaria*	4	7 (7, 0)
Triazole	*M*. *rotundata*, *O*. *bicornis*, *O*. *cornifrons*, *O*. *lignaria*	8	8 (6, 2)

For the purposes of this review, an investigation is defined as one or more PECO-relevant outcomes resulting from pesticide exposure (i.e., an investigation reporting multiple outcomes equates to one investigation).

*Individual publications may have investigated more than one species of solitary bee.

**Count excludes investigations that did not clearly specify if the test compound was technical grade or a commercial formulation.

**Table 5:** Herbicides studied available solitary bees. The number of investigations that used a formulation or technical grade active ingredient alone are shown in parenthesis.

Herbicide class	Bee species	Number of publications	Number of investigations (in formulation, technical grade)
Chlorinated phenoxy acid	*O*. *bicornis*	1	1 (0, 1)**
Phosphonate	*O*. *bicornis*	1	1 (1, 0)

For the purposes of this review, an investigation is defined as one or more PECO-relevant outcomes resulting from pesticide exposure (i.e., an investigation reporting multiple outcomes equates to one investigation).

**Count excludes investigations that did not clearly specify if the test compound was technical grade or a commercial formulation.

**Table 6:** Mixtures studied in solitary bees. The number of investigations that used a formulation or technical grade active ingredient alone are shown in parenthesis.

Mixture	Bee species	Number of publications*	Number of investigations (in formulation, technical grade)
Carbamate insecticide, pyridinecarboxamide fungicide	*M*. *rotundata*, *O*. *lignaria*	1	3 (3, 0)
Carbamate insecticide, pyridinecarboxamide fungicide, dicarboximide fungicide	*O*. *lignaria*	1	1 (1, 0)
Carbamate insecticide, pyridinecarboxamide fungicide, spray adjuvant	*M*. *rotundata*	1	1 (1, 0)
Carboxamide fungicide, strobilurin fungicide	*O*. *cornifrons*	1	1 (0, 0)**
Chlorinated hydrocarbon insecticide, organochlorine insecticide	*M*. *rotundata*, *N*. *melanderi*	1	2 (0, 0)**
Dicarboximide fungicide, surfactant	*O*. *lignaria*	1	1 (1, 0)
Dicarboximide fungicide, surfactant, fertilizer	*O*. *lignaria*	1	1 (1, 0)
Neonicotinoid insecticide, acylalanine fungicide, benzodioxole fungicide, ß-methoxyacrylate fungicide	*E*. *pruinosa*	1	1 (1, 0)
Neonicotinoid insecticide, imidazole fungicide	*O*. *bicornis*	1	2 (2,0)
Neonicotinoid insecticide, neonicotinoid insecticide	*O*. *bicornis*	2	3 (0, 2)**
Neonicotinoid insecticide, neonicotinoid insecticide, triazole fungicide	*O*. *bicornis*	1	1 (0, 1)
Neonicotinoid insecticide, phosphonate herbicide	*O*. *bicornis*	1	1 (1, 1)
Neonicotinoid insecticide, pyrethroid insecticide	*O*. *bicornis*	4	7 (6, 0)**
Neonicotinoid insecticide, triazole fungicide	*O*. *bicornis*	5	6 (2, 3)**
Organophosphate insecticide, neonicotinoid insecticide	*O*. *bicornis*	1	1 (0, 0)**
Organophosphate insecticide, phosphorothioate insecticide	*N*. *melanderi*	1	2 (0, 0)**
Organophosphate insecticide, triazole fungicide	*O*. *bicornis*	1	9 (0, 9)
Organosulfur insecticide, organophosphate insecticide, phosphorothioate insecticide	*M*. *rotundata*	1	1 (0, 0)**
Pyrethroid insecticide, adjuvant	*M*. *rotundata*, *N*. *melanderi*	1	4 (3, 1)
Pyrethroid insecticide, biopesticide	*M*. *rotundata*, *N*. *melanderi*	1	2 (2, 0)
Pyrethroid insecticide, surfactant	*M*. *rotundata*, *N*. *melanderi*	1	5 (5, 0)
Spray adjuvant	*M*. *rotundata*	1	1 (1, 0)

For the purposes of this review, an investigation is defined as one or more PECO-relevant outcomes resulting from pesticide exposure (i.e., an investigation reporting multiple outcomes equates to one investigation).

*Individual publications may have investigated more than one species of solitary bee and more than one mixture.

**Count excludes investigations that did not clearly specify if the test compound was technical grade or a commercial formulation.

#### Insecticides

Since publications often evaluated the same pesticide compound under different conditions (formulation, technical grade, dose-level, lab, semi-field, field) and in different species and sexes of bees, we counted the number of times a pesticide had been investigated as opposed to simply relying on the number of publications. Thus, within a publication, there may be multiple investigations of the effects of exposure to a pesticide or mixture on PECO-relevant outcomes. For the purposes of this review, an investigation is defined as one or more PECO-relevant outcomes resulting from pesticide exposure (i.e., an investigation reporting multiple outcomes equates to one investigation).

PECO relevant publications investigated the effects of 119 insecticides. The most studied insecticide groups were organophosphates (17 publications including 155 investigations of 46 insecticides), carbamates (7 publications including 81 investigations of 18 pesticides) followed by, neonicotinoids (27 publications including 36 investigations of 5 insecticides), and then pyrethroids (13 publications including 38 investigations of 14 insecticides). The remaining 14 pesticide groups studied had four or fewer publications. The most widely studied insecticide is clothianidin (27 investigations) followed by dimethoate (23 investigations), imidacloprid (22 investigations), aldicarb (22 investigations), carbaryl (11 investigations), and trichlorfon (11 investigations). Among the studies that reported the test compound configuration, more investigations evaluated technical grade active ingredient than formulated products (Table 3 & S3 Table in S1 File). However, just over half of the investigations did not specify which format was used.

#### Fungicides

PECO relevant publications investigated the effects of 10 fungicides from 7 different fungicide groups. The most commonly studied groups were triazoles (8 publications including 11 investigations of 3 fungicides) followed by dicarboximides (4 publications including 4 investigations of 1 fungicide) and then phthalimides (4 publications including 7 investigations of 1 fungicide) and benzimidazole (3 publications including 3 investigations of 1 fungicide). The most widely studied fungicides were propiconazole (8 investigations), captan (7 investigations), iprodione (4 investigations), and benomyl (3 investigations). Commercial formulations were studied most frequently followed by technical grade active ingredient and, in a few instances, the format tested was not specified (Table 4 & S3 Table in S1 File).

#### Herbicides

Based on our literature search, the effects of herbicides have only been studied in two publications. One herbicide studied, 2,4-Dichlorophenoxyacetic acid, is a member of the chlorinated phenoxy acid class of herbicides, has been evaluated as technical grade active ingredient in one investigation, and, the format was not specified in the second investigation (Table 5 & S3 Table in S1 File). Glyphosate, the other herbicide studied, has only been evaluated in a single investigation as formulated product (Table 5 & S3 Table in S1 File)

### Lethal effects of pesticides

Review of 23 publications identified 185 investigations of the lethal effects of pesticides on adult (179 investigations) and immature solitary bees (6 investigations) (Fig 3C; S3 Table in S1 File). Overall, available data provide insights into the effects of pesticides, primarily as single compound exposures, on lethality to adult bees and, to a much lesser extent, immature stages.

#### Adult bees

Pesticide-specific lethality can be quantified by determining the dose that kills 50% of the exposed population (i.e., LD_50_). In the context of exposure duration (acute vs. chronic) and route (i.e., oral or contact), LD_50_ values provide a mechanism to compare the relative potency of different pesticides and the sensitivity of different species to pesticides.

Contact and oral toxicity of 13 insecticide classes and 4 fungicide classes have been evaluated in adult and immature stages of *Osmia* species, *M*. *rotundata* and *N*. *melanderi*, primarily under laboratory conditions. The lethal effects of only 5 fungicides were evaluated in adult bees (S3 Table in S1 File). Out of 54 oral toxicity investigations, only 28 investigated the effect of chronic exposure on lethality (S3 Table in S1 File). Only 6 of 131 contact toxicity investigations evaluated chronic exposure (S3 Table in S1 File). Based on our literature search, there are no investigations of the effects of chronic contact exposure on lethality. Dimethoate, an organophosphate pesticide commonly used as a reference toxicant, has been evaluated in contact and oral toxicity tests as a technical grade active ingredient and in formulated products using *Osmia* spp., *M*. *rotundata* and *N*. *melanderi* [37–43]. However, the available dataset is too fragmented to thoroughly evaluate for reproducibility and cross-species sensitivity. Still, several publications [37, 44–48] and one literature review [49] concluded that *N*. *melanderi* is the most tolerant species, while *M*. *rotundata* is the least tolerant to a wide range of pesticides.

Very few studies have investigated the effects of pesticide mixtures on lethality (S3 Table in S1 File). Available investigations provide evidence for synergism between a fungicide and a neonicotinoid pesticide (i.e., fenbuconazole and acetamiprid and fenbuconazole and imidacloprid [40]), but not for a fungicide combined with an organophosphate pesticide (i.e., propiconazole and dimethoate [41]) or the combination of two pesticides (i.e., clothianidin and tau-fluvalinate [50]). Conflicting results have been published for the combination of propiconazole and clothianidin [41, 43]. Under the conditions studied, binary combinations of cyhalothrin with adjuvants and surfactants were shown to increase acute lethality in *N*. *melanderi*, but not *M*. *rotundata* [45].

### Immature stages

Developing solitary bees may be exposed to contaminants present in pollen/nectar provisions provided by the female and, depending on their life history, could also be exposed through other unique routes of exposure (e.g., soil and leaf parts). The typical approach applied to evaluating the effects of pesticides on brood development involves contaminating the nest provision and monitoring progression to the point of spinning a cocoon as an indicator of larval survival. This type of assessment has been performed for insecticide classes including benzoylphenyl ureas [51], carbamates [52, 53], neonicotinoids [54–59], organophosphates [60], pyrethroids [61, 62], as well as benzimidazoles [63], phthalimide, triazole and dicarboximide [64] fungicides. Using this approach, the acute topical LD_50_ for *M*. *rotundata* eggs and second/third instars treated with aldicarb, aldicarb sulfoxide or aldicarb sulfone ranged from 1,619–5,180 μg/g and 1,692–3,549 μg/g, respectively [53]. No other published LD_50_ values were identified by our literature review. However, decreased survival of eggs, larvae and pupae have been demonstrated in evaluations conducted with field-realistic concentrations under laboratory, semi-field and field conditions following exposure to novaluron [51], naled [60], trichlorfon [60] and spinetorum [61], but not with clothianidin [54, 56, 59] or imidacloprid [54, 58]. Fungicides, including benomyl, iprodione, captan, propiconazole and copper, have been shown to increase larval mortality [63, 65], but kresoxim-methyl had no effect on larval survival when tested by direct application of 1 μL of the field dose to the larval provision [65]. No available publications evaluated the effects of mixtures on immature development stages. A complete list of investigations can be found in (S3 Table in S1 File).

### Sublethal effects of pesticides

Determining the acute toxicity of pesticides is undoubtedly important for informing risk assessment. However, acute toxicity data only capture a small component of the overall toxicity profile of a pesticide, and it is widely recognized that sublethal effects on bee behavior and physiology are critical to consider [66, 67].

Sublethal investigations have been reported in 48 publications describing 292 investigations, including 264 of field-realistic exposure levels (as defined by the authors), 4 investigations of non-field-realistic exposure levels and 24 investigations that did not specify field relevance (Fig 3C). These investigations evaluated several important effect endpoints that fall into three subcategories (i.e., foraging and nesting activity, larval development and emergence from overwintering, and immunocompetence). Overall, this body of work is challenging to interpret because the limited number of investigations available are distributed across seven species of solitary bees and are further divided by the type of study (laboratory, cage, semi-field and field studies) and the test chemical(s) evaluated. Investigations of sublethal effects have been performed primarily in *M*. *rotundata* and to a much lesser extent in *Osmia* spp., *E*. *pruinosa*, and *N*. *melanderi*.

#### Foraging and nesting activity

Sublethal effects of single compound insecticide exposure on foraging activity [57, 68–70], flight [57, 71–74], and food consumption by adults [75–77] have received some research attention. Investigations focusing on aspects of nesting activity including the number of nesting bees [60, 78], number of brood cells produced [51, 52, 57, 60, 62, 69, 74, 78–80], number of nests/female [45, 57, 70], and the number of days spent nesting have been published. The effects of exposure to pesticide mixtures on foraging activity [55, 68–70, 81], flight [71, 73, 81], food consumption by adults [75, 76, 82] and nesting [55, 68–71, 73, 81, 83, 84] have also been investigated. Generally, effects observed depend on the pesticide(s) studied, and, since there are limited investigations, clear trends are difficult to establish.

#### Larval development and emergence from overwintering

Sublethal effects on immature development stages (i.e., eggs, larvae, pupae) and eclosion following exposure to pesticides have been investigated in studies involving *Osmia* spp., *M*. *rotundata*, but not *N*. *melanderi* (S3 Table in S1 File). No investigations have been performed on the potential for fungicides to induce sublethal effects on larval development and emergence from overwintering. Most insecticide classes have only been evaluated in a single investigation. Larvae are the most frequently evaluated developmental stage and include assessments of the number of cocoons [53, 57, 78], time to cocoon completion [54, 61, 74, 85] and cocoon darkening [54], and food conversion [56, 85, 86]. Pesticide effects on emergence and eclosion are also commonly studied with an emphasis on emergence success [52, 57, 61, 62, 70, 74, 80, 85, 86] and time to emergence [54, 58, 87]. Effects of pesticide exposure on offspring size [52, 54, 56–58, 61, 78, 80] and sex ratio [52, 57, 59–61, 70, 78, 80, 85, 87] have also received some research attention. Only four publications investigated the effects of pesticide mixtures on the number of eggs and larvae, larval mortality, overwintering survival and sex ratio [55, 70, 73, 81, 88].

#### Male reproductive physiology

Effects on male reproductive physiology (i.e., sperm quantity, sperm viability and total living sperm) has only been investigated in a single publication [87]. Additional studies on male reproductive fitness are needed.

#### Immune system effects

A strong immune system is important for protecting solitary bees from pathogens. Pesticide exposure, which is widely believed to be a driving factor in bee population declines [24], has been shown to impair the bee immune system [89, 90]. However, only one investigation has been published on the effects of pesticide exposure on solitary bee immunocompetence [77]. Hemolymph collected from male *O*. *bicornis* exposed to thiacloprid contained fewer hemocytes and was reported to be less effective at killing bacteria than hemolymph collected from non-exposed males and females. Melanization and wound healing were not affected by thiacloprid treatment under the conditions of this investigation. This work provides new insights into the functional consequences of exposure to a neonicotinoid pesticide and also highlights an area lacking adequate data.

*Pesticide effects on wild bee populations*. Solitary bees are the largest group of bees and are valued for their pollination services in both managed and natural landscapes. Therefore, there is a need to study the potential for pesticide use to adversely affect the richness and abundance of wild bee species, including solitary bees. However, limited investigations have been conducted to address these concerns. Only five relevant wild bee population investigations were identified in our literature search (S4 Table in S1 File). All of these investigations monitored wild bee abundance in the context of agricultural landscapes treated with pesticides. Only one of these investigations limited treatments to a single formulation during the sampling period [83]. The remaining investigations either obtained pesticide applicator use records [10] or more simply assayed for a specific subset of pesticides (i.e., 6 neonicotinoids) [91] to identify pesticides the bees may have been exposed to. None of these investigations directly measured pesticide levels in pollen and nectar collected by solitary bees or levels of pesticides in solitary bee bodies. Rundlöf and colleagues [83] did, however, determine the pesticide content of honey bee nectar and pollen collected from the same site and verified that *O*. *bicornis* brood cells contained pollen from oilseed rape plants.

## Evidence summary

**Fig 6:**
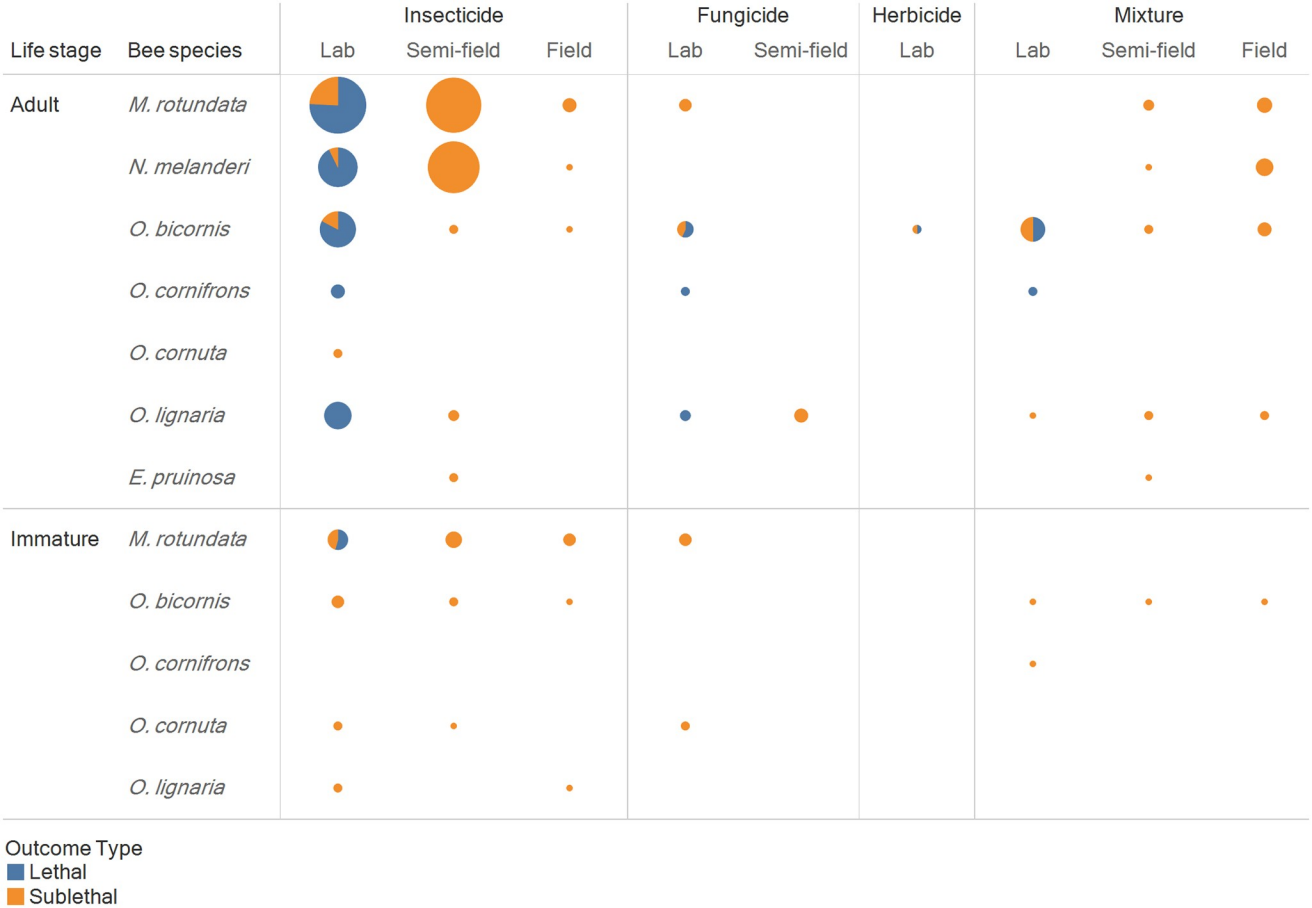
Evidence map of pesticide types and experimental conditions. Distribution of investigations across pesticide types and experimental conditions for adult and immature solitary bees. Colors correspond with investigations of lethal and sublethal outcomes (blue = lethal; orange = sublethal). Bubble size reflects the total number of investigations, ranging from 1 (smallest bubbles) to 87 (66 investigations of lethal and 21 investigation of sublethal effects of insecticides in adult *M*. *rotundata* under laboratory conditions); individual publications often investigate both lethal and sublethal outcomes.

**Fig 7:**
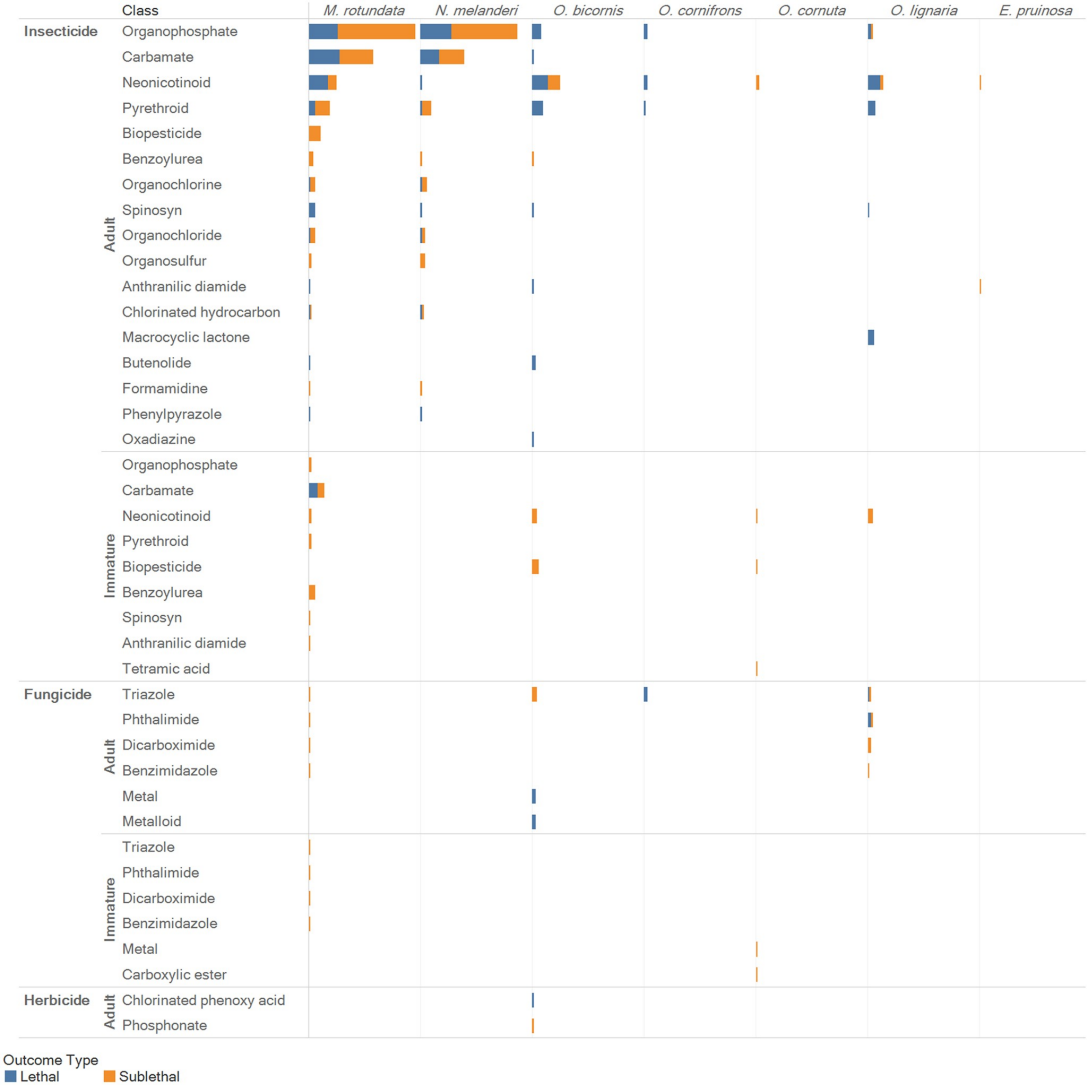
Evidence maps for each pesticide type broken down by pesticide class. Distribution of investigations across solitary bee species, life stage, and class of insecticide, fungicide, and herbicide. Colors correspond with investigations of lethal and sublethal outcomes (blue = lethal; orange = sublethal). Stacked bar size reflects the total number of investigations, ranging from 1 (smallest bars) to 71 (19 investigations of lethal and 52 investigations of sublethal effects of neonicotinoid insecticides in adult *M*. *rotundata*); individual publications often investigate both lethal and sublethal outcomes.

The effects of exposure to pesticides including insecticides, fungicides, herbicides mixtures of pesticides on solitary bee health are summarized in Figs 6 and 7. Review of the database revealed that 1) *M*. *rotundata*, *and N*. *melanderi* are the most commonly investigated solitary bee species 2) insecticides are the most frequently investigated type of pesticide, 3) herbicides are highly understudied, 4) of all pesticides, organophosphates (most common), carbamates, neonicotinoids, pyrethroids, and are the most frequently investigated, 5) irrespective of the type of pesticide, sublethal effects are more frequently investigated than the lethal effects of pesticides, 6) investigations performed on immature life stages are far outnumbered by investigations conducted on adults, and 7) relatively few investigations focus on exposure to mixtures of pesticides. Interpreting and applying the database to support pesticide risk assessment is further hampered by dilution across bee species, lack of complementary laboratory work and paucity of replicated investigations.

## Discussion of knowledge gaps

While there are data demonstrating adverse effects on the health and vitality of solitary bees following exposure to pesticides, there are several areas for which critical data are absent. The most critical data gaps are discussed here.

### Field-realistic doses

To better understand the nature of the dose(s) evaluated in solitary bee pesticide exposure-effects studies, we reported whether, or not investigators defined the concentrations they tested as field-realistic. While the practice of exposing adult solitary bees to pesticide prepared at the field application rate is an accepted practice, applying this approach to immature solitary bees warrants additional consideration. Irrespective of the field relevance of the concentration tested, applying droplets of pesticide directly onto eggs and larvae is not a field-realistic exposure scenario. Spiking food provisions with pesticide can provide information about the effects of pesticide exposure on immature life stages. However, it should be kept in mind that one provision mass may be collected from several contaminated flowers, each with a different level of pesticide contamination. Also, under field conditions, the pesticide is likely to be distributed throughout the provision, not isolated to one small focal area of the provision. Consequently, consumption of the pesticide by the developing larva will likely occur over a period of several days or even weeks. Further complicating matters, the floral composition and microbial content of the mass provision may affect nutritional quality and ultimately larval performance in toxicity studies. As these points illustrate, conducting and interpreting pesticide exposure studies involving immature solitary bee life stages is a complex endeavor. To maximize the value of these studies, we recommend selecting doses based on levels of the target pesticide detected in environmentally relevant matrices.

### Pesticide exposure

At least two types of data are needed to understand the impacts of pesticide exposure on solitary bee health: data on exposures as they occur in the environment; and data linking exposures to adverse health effects. Presently, data on pesticide exposures as they occur in the environment are limited. For example, additional studies are needed to better understand how soil and leaf parts contribute to pesticide exposure for some solitary bee species [1, 33, 92]. Data on nectar and pollen consumption for solitary bee larvae are limited and non-existent for adults [1] and should receive attention from researchers. However, seven publications examined the pesticide content of larval provisions collected under either semi-field or field conditions [51, 53, 57, 59, 71, 73, 81] and two publications under laboratory conditions [55, 76]. Six of those publications assayed *O*. *bicornis* provisions. Four of these publications confirmed that larval provisions collected from oilseed rape sowed from neonicotinoid dressed seeds contain low levels of the test neonicotinoid; and, in the case of an investigation involving thiamethoxam, a neonicotinoid metabolite (i.e., clothianidin) [57, 71, 73]. One investigation was performed for *M*. *rotundata* and *N*. *melanderi*. Food intake estimates coupled with real-world pesticide level data in food and from other relevant exposure routes (i.e. soil, leaf parts) are essential to assembling exposure estimates that are needed to support risk assessment.

Data on the types and quantities of pesticides in and on solitary bee bodies are also highly limited. Only one study investigated the effects of exposure through soil on solitary bee survival and nesting success [78]. In this study, nesting activity and the number of brood cells produced/day were significantly reduced, and the sex ratio was skewed towards males [78]. In addition, pesticide residues in mud collected from *O*. *bicornis* nests has been quantified in two investigations [81, 93]. Bi-monthly trap collections of solitary bees from grasslands and wheat fields isolated residues of 19 pesticides and degradates ranging from 1 to 310 ng/g per each chemical in 54 specimen samples [94]. In a similar study, 20 out of 282 collected bee specimens (including seven genera of solitary bees) from a variety of landscapes contained neonicotinoid compounds [95]. These data provide some context for interpreting experimental data and support the need for a higher understanding of the effects of complex mixtures of pesticides.

### Mixtures toxicology

Fifty investigations evaluated the effects of combinations of pesticides and pesticides combined with adjuvants on adult solitary bees while only three investigations evaluated effects on immature stages (i.e., larval and pupal survival, eclosion, and sex ratio). No investigations of mixtures have been performed on *N*. *melanderi* or immature stages of any solitary bee reviewed here. While insights have been gained from these investigations, available data are too limited to draw concrete conclusions. Some investigators might argue that toxicity data for individual mixture components are necessary to support risk assessment. However, this level of information is unlikely to be available for all mixtures and, as has been demonstrated [40, 43], mixtures may not produce the same magnitude of effect as single compound exposures (i.e., additive toxicity) and may even induce new sublethal effects. So, while it may be ideal to have comprehensive data on single compound exposures, additional investigations with field-relevant mixtures of pesticides and adjuvants are needed to support risk assessment.

### Reproductive success

Protecting female solitary bees and their ability to produce viable offspring is of paramount importance. The existing database for pesticide effects on fundamental elements of solitary bee reproduction (i.e., reproductive fitness, brood development and overwintering success) is inadequate with incomplete data for representative solitary species (e.g., no investigations with *N*. *melanderi* and only one investigation for *E*. *pruinosa*). Furthermore, although fungicides are widely applied in environments relevant to solitary bees, the effects of fungicides on brood development are limited to only two publications [63, 65] and, none have evaluated effects on overwintering survival. To that point, investigations focusing on female reproductive fitness, brood development, overwintering survival and the ability of the next generation to successfully mate and complete viable nests are needed. Recognizing that significant variation in the number of nests/female, number of brood cells/nest, sex ratio and offspring mortality have been reported under ideal conditions and that nesting parameters are influenced by foraging abundance and period of nesting activity [96–99], data derived from investigations of this nature must be carefully interpreted.

#### Pesticide metabolism

Even though biotransformation of pesticides is known to play a key role in organism responses to pesticides, only two investigations in solitary bees have been published on this important process. By sequencing the *O*. *bicornis* genome, Beadle and colleagues [100] showed that *O*. *bicornis* lacks the CYP9Q subfamily of cytochrome P450 enzymes known to detoxify the N-cyanoamidine neonicotinoids including thiacloprid. Despite not possessing the CYP9Q enzyme subfamily, thiacloprid exhibited low acute toxicity in *O*. *bicornis* because, as the authors discovered, *O*. *bicornis* possesses the CYP9BU subfamily that is able to detoxify thiacloprid thereby protecting the bees. *M*. *rotundata*, which lacks both CYP9Q and CYP9BU subfamilies, is >2,500 times more sensitive to thiacloprid and 170-fold more sensitive to flupyradifurone than the other solitary bee species evaluated [101]. Additional work is necessary to determine if *M*. *rotundata* is differentially sensitive to other pesticides. Furthermore, because it is now clear that cytochrome P450 enzymes that are responsible for detoxifying specific pesticides are not present in all solitary bees, genome sequencing may provide an alternative mechanism for achieving protection goals.

### Risk assessment considerations

Reliance on honey bees as the model organism for risk assessment performed on other bees is being questioned. Regulatory guidance is catching up with the need to evaluate adverse effects of pesticides on solitary bees. The European Food and Safety Authority (EFSA) and the U.S. Environmental Protection Agency (US EPA) have recommended a tiered risk assessment scheme that takes solitary bees into account [102, 103]. EFSA has also called for the development of solitary bee-specific toxicity tests [102].

There is an ongoing international movement to develop solitary bee-specific methodologies for evaluating the effects of acute and chronic pesticide exposure. Compiling available publications on solitary bee larval testing, Eeraerts and colleagues contend that the *Osmia* genus should serve as the model for developing specific toxicity test protocols for solitary bees [104]. As a result, current efforts to build testing methods are centered on commercially available *Osmia* species and have led to the development of an acute contact testing method for solitary bees [105]. International ring tests to confirm intra- and inter-laboratory reproducibility are ongoing. Progress has also been made in the development of techniques for rearing *Osmia* larvae *in vitro* [86, 106]. While this progress is notable and necessary, it is important to remember that *Osmia* ecology and life history are not representative of all solitary bees. For that reason, species that incorporate leaf parts into their nests (i.e., *M*. *rotundata*) or excavate below-ground nests (i.e., *N*. *melanderi*, *E*. *pruinosa*) may be vulnerable to additional pesticide exposure routes resulting in cumulative exposure to larger quantities of pesticide over time [1]. Coupling the potential for increased exposure levels with evidence for differential sensitivity to pesticides [22, 38, 107] leads us to recommend completion of studies conducted in parallel to clarify the meaningful differences between species and, thus, inform the future need for conducting these laboratory tests.

To maximize the utility of comparative studies and other investigations, study designs must incorporate appropriate experimental controls (i.e., vehicle-treated group and control toxicant-treated group). To that point, it is noteworthy that only 1 of the 65 PECO-relevant publications described herein included a control toxicant-treated group in the study design. Reference toxicants are integral to data interpretation because they provide a mechanism to detect variability, identify sources of variability (i.e., test compound batch, test animal source and husbandry practices, analyst performance) that could reduce reproducibility and thus strongly influence data interpretation. Furthermore, to facilitate cross-species extrapolation, doses should be reported on a per μg bee basis when conducting studies using adult bees [21].

As an alternative to traditional laboratory tests and in recognition of the laborious task associated with conducting tests in multiple species, some investigators are calling for increased use of *in silico* tools to support risk assessment [108]. Under this approach, the honey bee and the associated well-established tools for assessing toxicity would still form the nucleus of the risk assessment process. Supported by honey bee data, the effects of pesticide exposure on solitary bees could be predicted using *in silico* tools that account for differences in ecology, life history, pesticide metabolism, pesticide sensitivity. *In silico* approaches simulate field-realistic scenarios that could promote achievable protection goals. Presently, SOLBEE is the only solitary bee-specific *in silico* model available [109]. It is important to note that this model was not designed for use in pesticide risk assessment. The process for developing a tool to model solitary bee population dynamics may be streamlined by adapting existing honey bee models. However, constructing a simulation model for use in evaluating the likelihood of adverse effects on solitary bees from exposure to a pesticide or pesticides requires considerable empirical evidence to support regulatory decision making. As this review has highlighted, the current knowledge base has many gaps that could limit development of these models. Thus, it is imperative that a set of standardized, core toxicity testing methods be developed for assessing the potential effects of pesticides on representative solitary bee species. Not only will these data be useful for directly comparing pesticide sensitivity across species of bees, the data derived are essential for advancing solitary bee toxicity testing to the level of simulation modeling that will ultimately streamline future evaluations.

## Conclusions

The importance of solitary bees to natural and managed environments cannot be ignored. However, the effects of pesticide exposure on solitary bees remain poorly understood. This systematic scoping review identified and organized available publications to create an evidence map for the effects of pesticide exposure on solitary bees and to identify critical research gaps. Although there are clear data gaps, notable progress in developing laboratory toxicity tests has been made. The benefits of developing standardized toxicity testing methodologies go beyond short-term gains in the number of tests performed. These tests have the potential to feed into the development of *in silico* tools that could streamline future evaluations essential to achieving protection goals.

## Supporting information

S1 File (XLSX)
